# Heated Tobacco Products: Emerging Health Outcomes and Insights into Oxidative Stress and Pro-Inflammatory-Driving Mechanisms

**DOI:** 10.3390/antiox15010008

**Published:** 2025-12-20

**Authors:** Camilla Morosini, Fabio Vivarelli, Moreno Paolini, Donatella Canistro

**Affiliations:** Department of Pharmacy and Biotechnology, Alma Mater Studiorum—University of Bologna, Via Irnerio 48, 40126 Bologna, Italy; camilla.morosini2@unibo.it (C.M.); moreno.paolini@unibo.it (M.P.); donatella.canistro@unibo.it (D.C.)

**Keywords:** heated tobacco products, electronic cigarettes, oxidative stress, respiratory system, cardiovascular risk, neurotoxicity, fertility

## Abstract

With over 8 million deaths annually, smoking remains one of the most impactful global public health burdens. Heated tobacco products (HTPs) offer smokers the possibility of inhaling nicotine by heating tobacco, resulting in a significant reduction in the levels of toxic and potentially toxic compounds released. However, a growing number of studies indicate that exposure to HTPs increases oxidative stress and triggers pathophysiological changes, mirroring cellular mechanisms well-documented in conventional cigarette smoke exposure. In particular, evidence from animal models suggests that the use of heated tobacco products (HTPs) may represent a risk factor for respiratory and cardiovascular diseases, as well as a potential contributor to reduced male and female fertility. The following review summarizes the current state of knowledge on HTP toxicity, focusing on both preclinical and emerging clinical data on the health impact of HTP use and the putative harm reduction aspects.

## 1. Introduction

Although there has been a significant decrease in the overall tobacco consumption in several countries, smoking still remains one of the main health issues worldwide, making smoking cessation a critical world health priority [1]. While pharmacological and behavioral approaches to smoking cessation show that less than 10% of patients successfully quit smoking for at least 12 months, the launch in the market of Electronic Nicotine Delivery Systems (ENDS) initially diverted many smokers wishing to quit to consider switching to the use of such devices, maintaining the typical gestures associated with traditional cigarettes. Initially, they were advertised as almost risk-free even in the absence of proper scientific evidence-based data [2] and it should not surprising that their popularity has grown dramatically especially among young people [3]. To date, the most widely distributed devices can be divided into two main categories: “liquid e-cigs” (electronic cigarettes; e-cigs) that include an atomizer that vaporizes a liquid composed of vegetable glycerin (VG), polypropylene glycol (PG), with the possible addiction of nicotine and flavorings, and the “Heat-not-Burn systems” (HnB) or “Heated Tobacco Products” (HTPUSERS), which heat tobacco sticks through contact with an electronic heating metal blade [4]. The latter are of particular interest considering the Food and Drug Administration (FDA) authorized their marketing as Modified-Risk Tobacco Products based on the considerably lower levels of potential carcinogens and toxic chemicals that can harm the respiratory or reproductive systems [5]. It is important to mention here that a considerable number of independent studies confirms that these devices release lower concentrations of toxic compounds; however, here the main question is if that reduction can be sufficient for considering them a valid aid for people who wish to stop smoking, or who are trying to cut down as the UK National Health System (NHS) guidance recommends [5]. Numerous basic studies highlighted how exposure to ENDS mainstream aerosol could be associated with a higher risk for chronic non-communicable diseases, including impacts on the cardiovascular system, airways inflammation, lung damage, impairment of gonadal function, and an increased risk of cancer [6]. At the same time, there is insufficient evidence to support the efficacy of ENDS for smoking cessation [7].

Over the years, nicotine use has declined in U.S. teens as a result of intense tobacco control efforts targeting this age group. However, the recent popularity of e-cigarettes has been complicating efforts to further reduce nicotine use, and vaping use rose dramatically among teens [8]. Data from some observational studies showed how ENDS consumers, especially young vapers, are more likely to become HTP users [9].

Further, it was recently discussed that regardless of youths’ intentions, those who used e-cigarettes were 4.6 times more likely to use cigarettes and become tobacco smokers one year later, showing how current e-cigarette users were five times more likely than non-current users to become cigarette users [10,11].

The premise that the introduction of electronic cigarettes would constitute an important tool to promote smoking cessation is not currently supported by concrete evidence. On the other hand, emerging studies are exploring and characterizing the toxicological risks associated with these devices; this aspect becomes even more significant if not counterbalanced by a hypothetical long-term benefit, such as abstaining from tobacco products.

The present review will specifically focus on HTPs, which are among the most rapidly spreading nicotine delivery systems in Western countries, particularly among younger individuals. It will present the most relevant findings concerning their impact on the respiratory, cardiovascular, and reproductive systems, discussing the underlying pathogenic mechanisms reported in the literature, paying particular attention to the pro-oxidant and pro-inflammatory effects associated with HTP use.

## 2. ENDS Devices as a Harm-Reduction Strategy for Smokers: Their Evolution and Key Regulation Acts in the USA and Europe

This chapter aims to provide an overview of the introduction and development of electronic nicotine delivery systems (ENDS), describing their main functional characteristics and highlighting the key milestones that have shaped their current regulatory status and commercial use. Although the present manuscript focuses specifically on heated tobacco products (HTPs), it is important to offer the reader a broader perspective on the evolution of these devices in order to facilitate a clearer understanding of the current state of the art.

The term “electronic cigarette” and its related nicknames (e-cigarettes, e-cigs, vape pens) are beginning to spread and attract the attention of smokers or former smokers who see these new devices as a way to consume nicotine, maintaining the typical gestures associated with traditional cigarettes, but without the well-known health risks associated with tobacco product consumption. In this regard, the advertising campaigns that accompanied the launch of these products have certainly played a key role in the success of these devices. E-cigarettes were initially promoted as a risk-free aid to quit smoking. In the following years, some studies reported no clear evidence that e-cigarettes are an effective smoking cessation aid, while a growing and convincing evidence suggests that they can act as a gateway to cigarette smoking [12]. Alongside the increasingly widespread distribution of these products, they are beginning to undergo a true evolution in order to offer the consumer a more varied vaping experience, capable of attracting an ever-growing number of people, including younger individuals.

E-cig devices are mainly composed of a battery, an e-liquid mixture cartridge, and an electronic heating atomizer equipped with a coil to vaporize the e-liquid mixture to create an aerosol. The e-liquid typically contains propylene glycol (PG), vegetable glycerin (VG), nicotine, flavors, and other additives [4]. Although the main technical features have remained largely unchained over the time, the market witnessed a marked change, moving from the first generation devices, designed to mimic the appearance of combustible tobacco cigarettes, often called “cig-a-like”, to the second generation characterized by a much-larger-capacity battery and cartridge than the first generation, until reaching a third generation of devices that shares no commonality with conventional cigarettes in appearance and, most importantly, allowing the users to adjust voltage, atomizer power, and the resistance of the coil in terms of Ohm. Voltage and the resistance adjustment applied to the atomizer, as well as customization of the liquid composition by modifying the ratio of VG and PG, enable a high degree of personalization of the vaping experience; however, it also results in considerable variability in the concentrations of emitted toxicants, and consequently in the overall health impact [4].

The newest generation of e-cigarettes is a compact capsule-like device, also known as “Pod Mods”, which typically utilizes nicotine salts rather than free-base nicotine as in the previous generations. Next to these products, electronic devices have been developed to heat tobacco via conduction, allowing the user to inhale vapors produced by heating rather than by combustion. These brand-new devices were successfully distributed under the name of heated tobacco products (HTPs) or “heat-not-burn” e-cigarettes. With the possibility to fully release the active substances in tobacco by heating it instead, HTPs not only meet people’s demand for tobacco products, but they are also strongly advertised as lower-risk products [13]. Although HTPs are certainly influenced by factors such as the specific processing of the tobacco used and the presence or absence of flavorings, the number of variables involved in HTP systems is significantly lower compared to e-cigs, enhancing the reproducibility of specific datasets.

**Figure 1:**
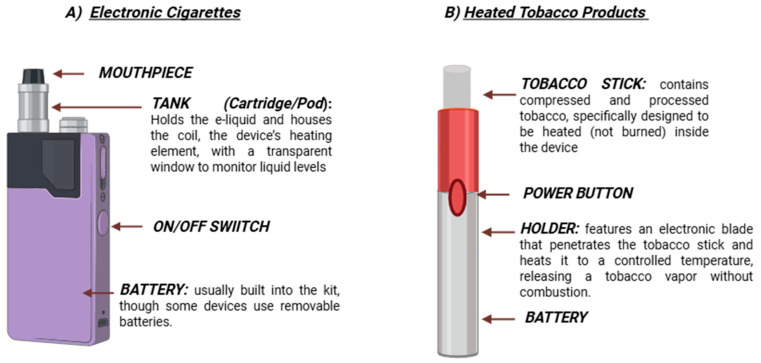
Schematic representation of e-cigs and HTPs.

Figure 1 summarizes the main differences between HTPs and liquid e-cigs.

Comparison between electronic cigarettes (A) and heated tobacco products (B). Electronic cigarettes consist of a tank (cartridge/pod) that contains e-liquid and a heating coil, powered by an integrated or removable battery, to generate an aerosol inhaled through the mouthpiece. Heated tobacco products, instead, employ specially designed tobacco sticks containing processed tobacco, which are heated by an electronic blade within the holder to a controlled temperature, releasing a nicotine-containing aerosol without combustion. These differences underline their distinct modes of operation and contribute to the toxicity associated with their use. Created in BioRender. Morosini, C. (2025) https://BioRender.com/cfts9hp (Academic License).

From the point of view of the key regulations on the US market, it is possible to outline the main regulatory stages as follows:

- In 2008, the FDA claimed these products fell under its jurisdiction and required their approval before they could be sold.
- In 2011, the FDA drafted a guidance document asking any company planning to, or already having, brought e-cigarettes to market to submit documentation of substantial equivalence if the product was similar enough to a tobacco product already on the market, or a premarket tobacco product application (PMTA) if the product was substantially different.
- With the rapid growth of the electronic cigarette market and under the pressure from the scientific community, in 2016 the FDA published a new guidance known as Deeming Rule imposing additional restrictions on the sale of tobacco products by mandating age verification (including online), while also requiring manufacturers to register new products, submit PMTAs, disclose ingredients, and include health warnings on the packaging. In the same year, Philip Morris International (PMI) submitted an application to the FDA to approve the marketing of its new HTPs as a “Modified Risk Tobacco Product” (MRTP).
- In 2017, the FDA delayed the PMTA submission deadline until 2022. Already dealing with the rising youth vaping crisis and record numbers of adolescent vapers, the American Academy of Pediatrics put pressure on the FDA and suggested a ban on flavored products (except for tobacco, menthol, and mint).
- In 2018, the number of hospitalizations due to e-cigarette- or vaping-associated lung injury (EVALI) increased [14], and a new FDA guidance on e-cigarette flavors was introduced: the guidance emphasized prioritizing the ban on flavored cartridges or pod-based e-cigarettes to reduce their appeal to minors, though several exceptions were made (e.g., tobacco- and menthol-flavored pod-based e-cigarettes, flavored disposable e-cigarettes, and flavored e-liquids for refillable e-cigarettes). As a result, many flavored e-cigarette products remain available, contributing to a surge in disposable e-cigarette use among middle and high school students from 2019 to 2020 [15].
- On 7 July 2020, the FDA partially authorized PMI’s MRTP application. While it concluded that the data submitted showed that the device may reduce the release of harmful substances, it did not agree that it reduces the risk of disease and death, compared to smoking cigarettes, and so had failed to meet the higher standard of “risk modification”. European countries lay down rules for electronic cigarettes sold as consumer products by Article 20 of the Tobacco Products Directive (2014/40/EU). The Directive sets a maximum nicotine concentration and volume for cartridges, tanks, and nicotine liquid containers. E-cigarettes should be child-resistant and tamper-evident and have a mechanism that allows refilling without spillage to protect consumers. E-cigarette ingredients must be of high purity, and e-cigarettes should deliver the same amount of nicotine when puffed at the same strength and duration. Health warnings for e-cigarettes advising consumers that they contain nicotine and should not be used by non-smokers are mandatory. Packaging must also include a list of ingredients contained in the product, information on the product’s nicotine content, and a leaflet with instructions for use and information on adverse effects, risk groups, addictiveness, and toxicity. Promotional elements are not allowed on e-cigarette packaging, and cross-border advertising and promotion of e-cigarettes is prohibited.

The phenomenon of novel ENDS devices has evolved very rapidly, leaving regulatory stakeholders with limited time to define and manage their use. This has often led to confusion, particularly regarding the terminology used to refer to the different types of electronic devices. In the present review, the term *ENDS* will be used as a general descriptor for any electronic device available for purchase in retail outlets specializing in tobacco products, thus excluding all medical-grade electronic devices [9].

This work will specifically focus on modified-risk tobacco products (MRTPs) equipped with heat-not-burn (HnB) technology, which will hereafter be referred to as heated tobacco products (HTPs). It is important to emphasize, for the sake of clarity, that the data presented herein refer exclusively to HTPs and should not be extrapolated to other devices, even those with seemingly similar mechanisms of action [15].

The specific characteristics of each product category (e.g., liquid-based electronic cigarettes vs. HTPs) influence not only the user’s vaping experience but also the observed outcomes in both experimental models and clinical settings. For this reason, any comparative extrapolation must be approached with the utmost caution.

### Overview of a Heated Tobacco Product (HTPs) and “Liquid” Electronic Cigarette (E-Cigs)

HTPs are the last electronic cigarette model launched on the market; they use an external heat source to heat the tobacco at a temperature typically below 350 °C to release a flavor without tobacco combustion. HTPs are operated by a battery-powered heating device with a heating blade that is inserted into a stick containing reconstituted tobacco (HTP sticks). Alternatively, the tobacco stick can be heated from the outside through a cylindrical heated chamber [16]. HTP sticks come in different flavors (e.g., menthol, tobacco, chocolate, berry, citrus), as do e-cig liquids. Heating versus combustion is the basic operational difference between HTPs and traditional tobacco products. In 2022, global “liquid” e-cigarette sales reached 18.85 billion US dollars, while HTPs reached 32.38 billion US dollars, showing a more significant market share increase for HTPs compared to the older models [16,17].

The tobacco industry claims that due to this operational difference, HTP aerosol contains fewer toxic chemicals compared to traditional cigarette smoke and, hence, it is a safer alternative to conventional smoking. To date, several independent studies have confirmed the significant reduction in numerous harmful and potentially harmful substances. Table 1 reports a comparative range of harmful and potentially harmful constituents recorded in mainstream heated tobacco products and the smoke of traditional combustible cigarettes.

The above-mentioned studies highlight several crucial aspects, although a significant reduction in the hazardousness of HPHC, but at the same time, some harmful constituents of tobacco products derived from elements from pyrolysis and incomplete combustion of the tobacco, such as acetaldehyde, benzo[a]pyrene (BaP), and carbon monoxide, are still present in the HTP mainstream. Some authors consider these elements to be sufficient to characterize the HTP mainstream aerosol as “smoke” [24]. Data confirm the presence of known carcinogens such as BaP, even though in concentrations significantly lower compared to those typically observed in traditional cigarette smoke, and it represents a key point since HTPs do not fall under the “smoking products” category, and in some countries, they are not subject to the current regulations regarding the use of cigarettes in public places. However, principle 1 for implementing article 8 of the World Health Organization convention on tobacco control highlights that we should reject ideas that there is a threshold value for toxic effects from second-hand smoke [26].

Again, as effectively summarized by the studies of St Helen et al., often chemical characterization studies on HTP mainstream do not cover the entire list of HPHCs of the US Food and Drug Administration. More in-depth studies on the topic show that among the not reported compounds, some of them are at least two-fold higher compared to TC smoke. Table 2 reports reviewed data from the literature, and they indicate that beyond doubt, HTPs release lower concentrations of toxicants, but at the same time they expose users to comparable or even higher levels of certain products [27].

Alongside the carcinogen compounds such as polycyclic aromatic hydrocarbons (PAHs), aromatic amines, tobacco-specific nitrosamines, and phenolic products, the presence of tar also contributes to several oxidative agents through redox-cycling reactions, when its constituents encounter molecular oxygen in the human lungs. The by-products of redox cycling in tar, such as free radicals and other oxidative agents, are linked to cancer development and increased inflammatory response. In fact, for decades, the scientific community has spread the point that “patients smoke for nicotine and die for tar” [28]. The tar phase contains several relatively stable free radicals, such as a quinone/hydroquinone (Q/QH_2_) complex held in the tarry matrix. This Q/QH_2_ polymer may function as an active redox system by reducing molecular oxygen in the smokers’ lungs to produce O_2_^•−^, which can eventually form other free radicals, such as H_2_O_2_ and ^•^OH. The generated reactive oxygen species (ROS) fuel a vicious cycle in which smoking-related oxidative stress causes inflammation, which in turn, results in further generation of ROS, and potentially increases oxidative damage to macromolecules that may trigger carcinogenesis. The aim of this work is to summarize the current knowledge on the use of HTPs and their role as risk factors for oxidative stress and inflammation, as well as the impact of this phenomenon at the systemic level.

## 3. The Impact of HTP Consumption on the Respiratory Cardiovascular System Its Role in Airway Disease

Since the consumption of traditional tobacco products predominantly impacts the respiratory and vascular systems, this area of research has been the most extensively investigated in the international literature. Below, we summarize the most significant findings obtained through both in vitro and in vivo models, and a dedicated section will address the available clinical data. The aim of this chapter is to provide the reader with a comprehensive overview of preclinical findings, highlighting those that have been corroborated by clinical evidence.

### 3.1. Role of HTP Use on Airway Diseases: Results from Preclinical Studies

Increased oxidative stress induced by tobacco smoking is a well-recognized key driving mechanism involved in several respiratory diseases, including chronic obstructive pulmonary disease (COPD) and its comorbidities as asthma, cystic fibrosis, bronchiectasis, idiopathic pulmonary fibrosis (IPF), and pulmonary hypertension [29]. One of the first in vitro pilot studies showed how bronchial epithelial cells directly exposed to HTP mainstream were found to exhibit higher cytotoxicity and a lower metabolic activation compared to controls, although changes were milder compared to TC smoke exposure. Markers of inflammatory response were also measured, and interestingly, cytokine IL-1β and IL-6 were overexpressed in cells exposed to HTP mainstream, as is usually observed in smoking-induced diseases where oxidants trigger inflammation through the activation of damage-associated molecular patterns (DAMPs). Both HTP and TC exposure increased the binding activity of Nrf2 to antioxidant response elements and the expression of its downstream targets. Concordantly, HTP use induced oxidative DNA damage and significantly increased DNA strand breaks and chromosomal aberrations, eliciting a biological response very similar to that of TC in human epithelial pulmonary cells [30].

Further studies have explored in vivo the effects of HTP mainstream, confirming the increase in oxidative stress and the induction of the secretion of inflammatory cytokines, first in short-term exposure models [31] and then in sub-chronic exposure. Studies reported higher ROS concentration in lung biopsies, the rise of the main DNA, protein, and lipid oxidative damage markers, and again the activation of the downstream cytokines through MAPK and NF-κB pathways [32]. Interestingly, images from electronic microscopy showed that the lung parenchyma was markedly disrupted with granulocyte pulmonary infiltration and higher circulating blood neutrophils, typically observed in patients with stable COPD.

Focusing on glutathione, a well-known indicator of oxidative stress on lung tissue and alveolar macrophages, some studies reported that many macrophages had intracellular foaming, likely due to their activation following the HTP exposure. This observation was very similar to the lipid accumulation in alveolar macrophages observed in mice exposed to tobacco smoke, as well as e-cigarette mainstream aerosol [33]. A disruption of the homeostasis of lipid metabolism, such as lung surfactants, was observed, resulting in lung inflammation and immune system compromise, and similar effects are triggered by HTP exposure [31]. The effects of increased oxidative stress stimulate the production of inflammatory cytokines. Similar to what is observed in human alveolar and tracheal epithelial cells exposed to combustible cigarette extracts, data from cellular and in vivo models confirm the increased expression of the GM-CSF gene and macrophage inflammatory protein CCL3. This, in turn, can stimulate the alveolar macrophages to secrete pro-inflammatory cytokines and may be associated with COPD progression. Additionally, the up-regulation of CCL3 facilitates the recruitment of macrophages into the airways [32]. These data suggest that HTP use may trigger a mechanism whereby increased oxidative stress and the release of pro-inflammatory factors result in tissue damage, which can significantly contribute to the exacerbation of respiratory diseases such as COPD [34].

Comparing the effect of short-term exposure to HTPs versus TC smoke in mice, some results indicate that the total number of lung-infiltrating leukocytes was equivalent after exposure to both HTP aerosol or CS smoke, but it was significantly increased compared to air-exposed controls. Moreover, a significant increase in CD4^+^RORγt^+^ T cells was observed in both groups. The RORγt receptor plays a critical role in the differentiation of Th17 lymphocytes and is implicated in the pathogenesis of autoimmune disorders and inflammatory responses. These findings are consistent with those previously described, leading to the conclusion that both HTPs and conventional cigarettes induce comparable lung damage and pro-inflammatory alterations [35]. Supporting these findings, the increase in CXCL8 chemokine from human bronchial epithelial cells and primary human airway smooth muscle cells following HTPs, liquid e-cigarette or CSs, and the increased release of collagen 1 and fibronectin strengthen the hypothesis that e-cigarettes and HTPs, similar to classic cigarettes, can increase oxidative stress, inflammation, and airway reconstruction [36].

In contrast, the study by Wong et al. reported an accumulation of macrophages, neutrophils, and CD4^+^ and CD8^+^ lymphocytes in the lungs, along with significantly elevated levels of inflammatory mediators in the bronchoalveolar lavage fluid (BALF) of mice exposed to conventional cigarette smoke, but not in those exposed to HTP aerosols. Similarly, impaired lung function and emphysematous alterations were observed exclusively in the CS-exposed group [37].

### 3.2. Influence of Heated Tobacco Product (HTP) Exposure on Cardiovascular Function: Results from Preclinical Studies

**Figure 2:**
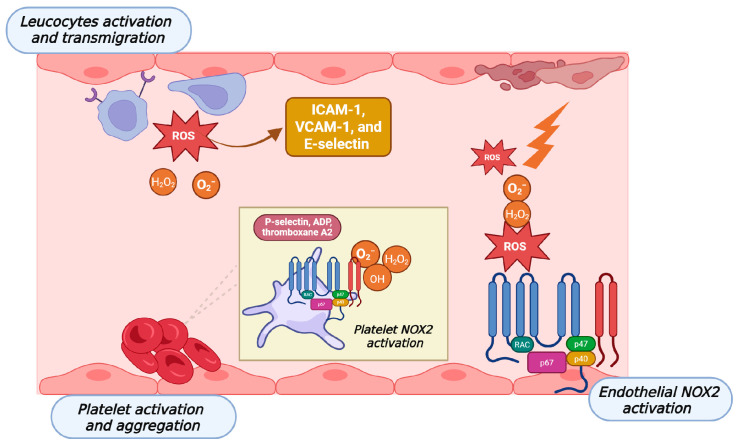
Shared redox-dependent mechanisms of smoking-induced endothelial dysfunction. The figure depicts the main pathophysiological processes driving smoking-induced endothelial dysfunction that are shared by conventional tobacco cigarettes, electronic cigarettes, and heated tobacco products. Exposure to smoke or aerosol increases reactive oxygen species (ROS) levels through both inhaled oxidants and enhanced endogenous production, primarily mediated by NADPH oxidases (NOX), including NOX2 in platelets and endothelial cells. The resulting redox imbalance, characterized by increased superoxide and hydrogen peroxide, promotes oxidative stress and reduces nitric oxide bioavailability. ROS-dependent endothelial activation induces the expression of adhesion molecules (ICAM-1, VCAM-1, and E-selectin), facilitating leukocyte activation and transmigration. In parallel, platelet NOX2 activation enhances platelet activation and aggregation through the release of pro-thrombotic mediators such as P-selectin, ADP, and thromboxane A_2_. Together, these oxidative and inflammatory pathways contribute to endothelial dysfunction, vascular inflammation, and thrombus formation, representing a common cardiovascular mechanism across different smoking and vaping products. Created in BioRender. Morosini, C. (2026) https://BioRender.com/9f8dafr (Academic Lincense).

The link between smoking and cardiovascular disease is well established; endothelial dysfunction is a critical indicator of cardiovascular damage and is considered an early predictor of cardiovascular events and prognosis in smokers. The pathophysiological processes driving smoking-induced endothelial dysfunction (SIED) are complex, as summarized in Figure 2. A pivotal role is played by oxidative stress, mainly generated by the accumulation of ROS, which diminishes the bioavailability of nitric oxide (NO). Interestingly, in smoking conditions, ROS comes from both the inhaled fraction and from endogenous cellular mechanisms. One of the main ROS-generating cellular pathways involves nicotinamide adenine dinucleotide phosphate oxidase (NOX), which transfers electrons from cytoplasmic NADPH to molecular oxygen, increasing the formation of O_2_^−^ and H_2_O_2,_ and reducing the nitric oxide (NO) reservoir, creating a state of oxidative stress. Studies on tobacco products indicate that smoke enhances the recruitment and activation of leukocytes by increasing the expression of adhesion factors such as ICAM-1, VCAM-1, and E-selectin in endothelial cells [38]. Furthermore, exposure to foreign toxicants such as those present in CS or HTP mainstream can trigger the secretion of various immune mediators, including cytokines from the pulmonary compartment, inducing a condition of systemic inflammation, thereby affecting the circulatory system and contributing to an increased risk of atherosclerosis [39]. Some evidence seems to confirm the pro-oxidative and pro-inflammatory effects of HTP exposure, in particular, an in vitro 3D cardiovascular co-culture model.

It was shown that HTPs, although to a significantly lesser extent compared to CS, lead to a glutathione depletion and an increase in inflammatory markers, along with higher monocyte adhesion following exposure. These results suggest a putative increase in cardiovascular risk through a pathway of oxidative stress, inflammation, and endothelial dysfunction [40]. More recently, in an in vitro model of monocyte adhesion using a vasculature-on-a-chip platform mimicking the tissue-resident macrophage-mediated endothelial activation, the authors showed how HTP exposure increased monocyte adhesion and the release of TNF-α and IL-1β due to the pro-inflammatory cytokine secretion from macrophages, indicating a risk of atherosclerosis, which, nonetheless, was found to be markedly reduced in comparison to exposure to CS [41].

Since phosphorylation of eNOS at Ser1177 positively regulates endothelial nitric oxide synthase (eNOS) activity and NO generation, the impact of HTPs on eNOS phosphorylation was explored. Some HTP tests disrupt eNOS and NO content, indicating that HTP consumption, mirroring the effect of CS, can increase the risk for endothelial dysfunction [42].

Some animal models on short-term or chronic exposure to HTPs showed alterations in endothelial function similar to those induced by traditional cigarette smoke. A single exposure to HTPs can rapidly impair endothelial function in rats comparably to smoke from a cigarette [43].

The preclinical data and the results from clinical trials available to date, despite the discrepancies among studies, seem to indicate that a complete switch from CS to HTPs confers benefits in terms of reducing markers of inflammation and oxidative stress, as well as improving key clinical parameters associated with cardiovascular risk. A key challenge lies in accurately assessing the risk associated with new or emerging HTP consumers. While studies clearly indicate a reduction in harmful and potentially harmful constituents (HPHCs) in the aerosol emitted by HTPs, emerging factors with potential toxicological relevance further complicate the picture—such as the presence of toxic metals released due to device wear and degradation. A recent review reveals that nickel and cadmium may contribute to oxidative stress, promoting DNA damage and contributing to the impairment of the endothelial cells’ function, along with interference with the ion balance affecting vascular tone and cardiac contraction [4]. Currently, for these reasons, and in the absence of long-term clinical data, all major heart international societies do not recommend the use of electronic or heated tobacco products as a means to quit smoking.

### 3.3. Data from Clinical Studies on HTP Exposure and Biological Effects: Biomarkers Associated with Cardiovascular and Respiratory Function

In this paragraph, the major clinical studies that have evaluated established exposure biomarkers will be analyzed in order to assess the role of HTPs in potential risk reduction compared to the use of combustible tobacco products.

To date, clinical trials aimed at investigating the impact of HTPs on human health are still scant, and for ethical reasons, mainly only cohorts of patients switching from the use of traditional tobacco products to HTP or HTP native users can be considered.

Table 3 provides the main clinical trials focusing on exposure and biological effects biomarkers related to oxygen delivery, genotoxicity, inflammation, oxidative stress, lipid metabolism, endothelial function, platelet activation, and cardiovascular and respiratory function.

The majority of currently available clinical studies suggest that HTPs may play a role in harm reduction if smokers completely switch to HTPs from combustible cigarettes. It has been shown that there is less exposure to toxic substances like carbonyls or ROS using HTPs, and these products also show better results in clinical trials with fewer exacerbations in COPD patients switching to HTPs compared to continuing smoking [73]. An important 3-year study compared patients with COPD who quit traditional tobacco smoking or significantly limited their use in favor of HTPs with patients of the same age and sex who continued smoking. A significant reduction in the number of annual exacerbations was found in patients using HTPs, and no significant changes were recorded in COPD patients who continued to smoke [44]. Given the improvements in exacerbation rates, respiratory symptoms, and overall health status, it was not surprising that patients in the HTP user group consistently experienced downstaging (i.e., improvement) of their COPD stages throughout the whole duration of the study. In this light, for COPD patients unwilling to quit smoking, switching to HTPs should be seen as a valuable solution to the challenging problem of smoking. However, many authors are still firmly convinced that the increase in knowledge base about the respiratory health risks and benefits associated with HTP use is mandatory before considering HTP use as a part of clinical guidelines, a key for better policymaking, and for better quality of information for end-users.

Acute eosinophilic pneumonia (AEP) is a rare but serious condition that leads to severe respiratory failure. Typically triggered by the inhalation of allergic antigens, such as chemicals and fungi, it counts among its primary causes the smoking habit. Also, in this case, the presumed “harmless nature” of HTPs is being questioned. Although clinical studies on the putative role of HTP consumption on new AEP occurrence are still scant, some case reports showed elevated inflammatory markers and eosinophilic lung disease, suggesting HTP use may trigger respiratory issues due to its particulate emissions [74,75]. The mechanism of how smoking causes AEP has not been clarified; however, some chemical substances contained in cigarettes could contribute to activating an allergic reaction and eosinophils. In pediatric populations, exposure to cigarette smoke, whether active or passive, poses significant health risks, particularly regarding long-term cardiovascular outcomes. The deleterious effects of secondhand exposure are extensively documented in the literature pertaining to the respiratory health of children. Tobacco smoke functions as a pro-inflammatory stimulus, contributing substantially to the pathogenesis of asthma, allergic rhinitis, atopic conditions, and obstructive sleep apnea syndrome. These respiratory disorders have been associated with elevated cardiovascular risk in adulthood, likely mediated by the persistence of systemic low-grade inflammation initiated during early life exposure. Lee et al. found that HTP use in adolescents correlated with asthma, allergic rhinitis, and atopic dermatitis, implying a possible role in immune sensitization [76]. Particular attention should be paid to adolescents since, using these products less heavily than adults, they could be exposed to concentrations of allergens that are sufficient to sensitize, and not sufficient to suppress the sensitization [75].

The levels of tobacco exposure-related biomarkers were improved following HTP use, compared to smoking TS. The favorable change in biomarkers related to smoke exposure, especially those linked to inflammation and oxidative stress, could potentially contribute to improved health outcomes. Changes in adhesion molecule-1 (sICAM-1) in serum, white blood cell (WBC), 11 dehydrothromboxane B2 (11-DTX-B2), and 8-epi-prostaglandin F2 alpha (8-epi-PGF2α) have been reported as associated with smoking-related diseases such as cardiovascular diseases. In the study by Ludicke et al., healthy smokers who switched to HTPs for 26 weeks reported how, among the eight biomarkers assessed—HDL-C, white blood cell (WBC), forced expiratory volume in one second (FEV1) post-bronchodilator expressed as % predicted (FEV1%pred), carboxyhemoglobin (COHb) in blood, total 4-(methylnitrosamino)-1-(3-pyridyl)-1-butanol (total NNAL), soluble intercellular adhesion molecule-1 (sICAM-1) in serum, 11 dehydrothromboxane B2 (11-DTX-B2), and 8-epi-prostaglandin F2 alpha (8-epi-PGF2α)—statistically significant improvements were observed in five of the eight co-primary endpoints after 6 months for patients who shifted to HTP consumption. This can indicate that HTP could be considered as an acceptable alternative when the smoking cessation clinical path fails [58]. However, some studies show how HTPs significantly increased inflammation, as indicated by elevated white blood cell counts and pro-inflammatory cytokines such as IL-6, IL-8, TNF-α, and CRP. This inflammatory response correlated with endothelial dysfunction, leading to increased arterial stiffness, as measured by pulse wave velocity and augmentation index [75]. The switching from traditional tobacco cigarettes to HTP use significantly reduces the carboxyhemoglobin concentration, but the white blood cell count, and data from plethysmography, such as airways resistance, specific airway resistance, and conductance, confirm a significant impact of HTPs on consumers’ health compared with the non-smoking cohort [77]. In an observational cross-sectional study, chronic use of HTPs was associated with impaired endothelial function, elevated oxidative stress, and enhanced platelet activation. In particular, the study highlights an increased level of soluble Nox2-derived peptide (sNox2-dp), indicating sustained NOX activation supported by the higher serum hydrogen peroxide (H_2_O_2_) levels, underscoring oxidative stress that may, in turn, lead to reduced NO bioavailability and, consequently, contribute to the progression of atherosclerosis through endothelial dysfunction. Interestingly, at comparable cotinine levels, a recent trial found a significant reduction in flow-mediated dilatation (FMD) with no significant difference between the HTP and CS smoker groups [60]. According to another recent trial, HTP smoking has shown an acute impact on systolic and diastolic myocardial function in healthy HTP consumers, comparable to that induced by traditional cigarette smoking [78].

In line, the results from a large metabolomic study indicate that HTP users present alterations in plasma glutamate and other biomarkers included in glutamate metabolism (ie, glutamate, arginine, ornithine, and citrulline) strongly associated with smoking. Multiple studies have indicated that glutamate levels are associated with the prospective development of cardiovascular diseases and may contribute to atherosclerotic plaque formation. Alterations in the glutamatergic pathway could partially elucidate the mechanisms underlying smoking-induced cardiovascular pathology [68].

Again, investigating the miRNA profile of chronic exclusive HTP users, the authors identified potential pathogenetic signatures, suggesting a significant impact of HTPs on circulating miRNAs [79] and the trial by Belkin et al., reported clinically elevated parameters of arterial vascular stiffness as a correlate for endothelial dysfunction associated with the HTP use [80] and this evidence is supported by data from a recent trial on healthy young adults showing how HTPs have immediate adverse effects on vascular function, resulting in increased arterial stiffness and platelet thrombus formation, known risk factors for the development of atherosclerosis [71].

Among children exposed to passive smoke from HTPs or CS versus unexposed subjects, a recent trial reported significant changes in sCD40L, soluble P-selectin, and the Total Thrombus Formation Analysis System (T-TAS) as markers of platelet activation and thrombus formation. The authors conclude that the exposure to HTPs promotes oxidative stress, endothelial dysfunction, platelet activation, and thrombus formation in children [60]. Notably, increasing attention has been directed toward the effects of “thirdhand” smoke exposure involving the persistent residual pollutants that settle on surfaces and can be re-emitted into the air over time.

Nevertheless, it should be emphasized that the biomarkers considered in the studies mentioned above reflect processes on the pathway to smoking-related diseases: their predictive and discriminative power has yet to be established, so larger and longer-term population-based studies are needed to further clarify these findings [73].

In conclusion, although the current evidence supports the use of non-combustible smoking alternatives such as HTPs, which show evidence of improved levels of both exposure and biological effects, some results are conflicting, and confirmatory data are needed, so this remains a fertile research area in the coming years.

## 4. Raising Evidence of the Effects of HTP Exposure on the Central Nervous System (CNS)

Since their introduction into the market, the majority of the studies have mainly focused on the respiratory and circulatory systems. Recently, the World Health Organization (WHO) stressed the urgency of filling critical knowledge gaps, particularly with regard to the potential effects of ENDS on the central nervous system (CNS) and the association between ENDS use and nicotine dependence, as well as its impact on brain development in fetal life [81].

Nicotine is a major contributor to the immediate increase in regional cerebral blood flow (rCBF) caused by smoking, and recent data from animal models confirm that both smoking cigarettes and HTP aerosol extracts increase the rCBF, disrupting cerebrovascular health, suggesting that the effects on cortical vessels, i.e., vasodilation, are mediated by nicotine-induced α4β2-nAChR activation, setting aside toxic non-nicotine constituents of these two extracts as potential mediators of this effect [82]. Although the magnitude of the effects triggered by the HTPs is smaller than that induced by the nicotine intake from cigarette smoking. These data are in line with those presented in the previous chapter and confirm the impact of HTPs on vascular systems, including the cerebrovascular one. To further explore the potential impacts on cerebrovascular health, a recent study focused on the effects of CS versus HTPs on microglial function under conditions of hypoxia/reoxygenation (H/R). Ischemic stroke, caused by the obstruction of cerebral blood vessels leading to reduced perfusion of brain tissue, remains a leading cause of mortality and long-term disability across Western countries. Neuroinflammation and oxidative stress are key mediators of secondary brain injury following ischemia, further highlighting the substantial health and socioeconomic burden associated with stroke-related morbidity and mortality [83]. Results from an in vitro model of H/R confirm that CS exposure under H/R conditions reduces microglia activation, an essential step in the neuroinflammatory response to brain injury. HTP exposure maintained cellular function, suggesting potentially lower cytotoxicity of HTPs. Both CS and HTP aerosols show an upregulation of RNA-binding proteins, indicating a potential adaptive response to oxidative damage modulating oxidative stress-related proteins, albeit through distinct mechanisms: HTP aerosol and nicotine preserved nuclear translocation of Nrf2, a key regulator of the antioxidant response, whereas cigarette smoke impaired Nrf2 activation—suggesting elevated oxidative stress and increased potential for cellular injury [84]. The differential activation of the Nrf2-mediated antioxidant pathway implies that HTPs may exert a lower oxidative burden compared to conventional cigarettes. However, the health impact and associated risk in individuals who are primary or only HTP users need further investigation.

HTP exposure significantly increases ROS production in the prefrontal cortex of adult rats, along with an increase in pro-inflammatory cytokine and the down-regulation of PPARα and PPARγ nuclear receptors as negative regulators of oxidative stress-induced inflammation [84]. Again, HTP exposure increased the level of 8-hydroxyguanosine, a reliable marker of DNA oxidation that is positively associated with smoking habit, xeroderma pigmentosum group C protein complex (XPC), and 8-oxoguanine DNA glycosylase-1 (OGG-1), two crucial proteins involved in nucleotide excision repair (NER) and DNA base excision repair (BER). The pro-oxidant and neuroinflammatory effects of HTP exposure were also observed in human microglial cells; however, the comparison with CS treatment confirms again that HTP use can be associated with a lesser degree of microglial toxicity, with reduced ROS production, lipid peroxidation, oxidative DNA damage, and mitochondrial dysfunction [85].

**Figure 3:**
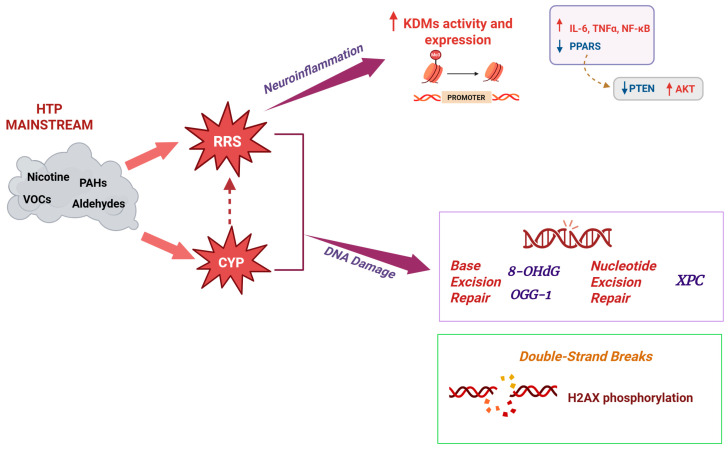
Effects of mainstream smoke from HTPs. Compounds present in HTP mainstream include numerous chemicals that can undergo CYP-mediated bioactivation to mutagenic or carcinogenic intermediates. Both this process and direct exposure to HTP aerosols contribute to the generation of reactive radical species (RRS), including superoxide and hydrogen peroxide. The resulting redox imbalance promotes neuroinflammation through epigenetic modulation of gene promoters by histone demethylases such as KDMs, which enhances the transcription of NF-κB–dependent pro-inflammatory mediators (IL-6, IL-1β, TNFα) and concomitantly reduces the expression of PPARs, key negative regulators of oxidative stress–induced inflammation. Although the precise mechanisms are not yet fully understood, an increase in both the activity and gene expression of KDMs has been reported after HTP exposure. In parallel, RRS-induced genotoxicity leads to multiple forms of DNA damage—such as oxidative lesions (e.g., 8-oxo-dG) and strand breaks—that activate DNA repair pathways. This is reflected by the increased expression of BER- and NER-associated proteins, including OGG1 and XPC, which are essential for counteracting the mutagenic consequences of oxidative and structurally distorting DNA lesions. Created in BioRender. Morosini, C. (2026) https://BioRender.com/z652ydp (Academic License).

Several studies suggest the relationship between lung stressors such as air toxicants and CNS, and the inflammation and oxidative stress play a partial mediating role in the lung–brain axis [86]. A growing body of evidence indicates that chronic inflammation, encompassing both systemic and neuroinflammation, is critically involved in the neuropathological progression of Alzheimer’s disease (AD) and in order to better explore this filed, in a recent study employing a mouse model mimicking prodromal AD, the authors observed a minimum impact on amyloid pathology; on the other hand, HTPs were found to affect gene modulations associated with the activity of neuropeptide hormones like arginine vasopressin, oxytocin, and galanin, a hormone known to modulate mesolimbic dopaminergic neurotransmission and associated with nicotine addiction [78] similarly to what previously observed in studies on CS [87]. Interestingly, early and progressive dysfunctions of the dopaminergic system from the Ventral Tegmental Area (VTA) have been described in AD [88] and recently, the exposure to HTP mainstream was found to increase KDM6B levels, along with downregulation of both PPARα and PPARγ gene expression in the VTA of exposed rats suggesting a putative mechanism associate with the pro-inflammatory effects [89]. Furthermore, the reduced expression of PTEN in exposed animals has already been documented in AD brains [90]. The increased AKT activity observed suggests a positive contribution of HTPs to neuroinflammation and virtually to AD progression, considering that a hyper-activation of GSK-3β due to AKT leads to tau hyper-phosphorylation, an important event in AD pathogenesis [91] (Figure 3).

Clinical data on the putative effects of HTP consumption on CNS pathologies are extremely scant. Noteworthy is an independent trial that surprisingly found that HTP-only users showed the highest proportion of anhedonia and depressed mood. CS-only users had the highest proportion of individuals who had trouble sleeping, while dual users had a higher proportion of those with fatigue and appetite problems [92]. These findings gain greater significance when interpreted in light of recent evidence showing that among adolescents, almost a third of those who had experimented with HTPs continued to use them [93], confirming that while HTPs may serve as a potential strategy for smoking cessation, they also pose a risk of acting as a gateway for non-smokers to initiate the use of these devices.

## 5. Emerging Data on HTP Use and Reproductive Health

Tobacco smoking still remains one of the leading causes of male infertility, due to the presence of nicotine, tar, carbonic monoxide, PAHs, and heavy metals, which are also partially present in HTP mainstream products. Spermatozoa are particularly susceptible to oxidative stress due to the high content of polyunsaturated fatty acids in membranes, the limited antioxidant capacity, as well as the ability of spermatozoa to generate ROS contributes to testicular inflammatory response affecting spermatogenesis and testosterone synthesis [94]. The deleterious effects of smoking on male gonadal function and fertility were extensively investigated [95]; however, the introduction of e-cigarettes has substantially heightened the scientific community’s interest in this area of investigation.

Tobacco consumption leads to exposure to over 100 carcinogens and mutagens, including PAHs, aldehydes, and heavy metals, which ultimately lead to DNA damage and testicular cytotoxicity, and some of them, as previously discussed, are still present in HTP mainstream despite concentrations still being significantly lower compared to CS. Although the studies on this topic are still scant, some animal models showed that parentally HTP-exposed male offspring mice exhibited changes in testicular morphology and decreased spermatogenesis, suggesting a significant impairment of male testicular function or delayed sexual maturation. In particular, the exposure during the organogenetic period leads to delayed spermatogenesis in juveniles and an increased incidence of abnormal seminiferous tubule morphology. These effects may be attributable to delayed sexual maturation or may result from a broader developmental delay. Surprisingly, the study reported no adverse effects of prenatal CS (3R4F) exposure on spermatogenesis or increases in the incidence of seminiferous tubule damage in male offspring [96]. These data are of particular interest because they represent one of the rare investigations showing a worsening effect associated with HTPs compared to CS. Although the concentrations of tobacco-specific toxicants in HTPs mainstream are significantly lower than those in CS, some constituents, such as 4-aminobiphenyl (4-ABP), for which sufficient evidence is available about its carcinogenicity, have been shown to cross the placenta, and it has also been shown that 4-ABP binds to fetal DNA [97]. This may offer a plausible explanation for the observed data, although the hypothesis has yet to be confirmed. Supporting data on toxic effects as a significant increase in oxidative stress markers—particularly those indicative of DNA damage—was observed in exposed animals. This was accompanied by the upregulation of NF-κB-dependent pro-inflammatory mediators, including TNF-α, IL-1β, IL-6, and COX-2. Additionally, the inactivation of key androgenic enzymes, such as 3β-hydroxysteroid dehydrogenase and 17β-hydroxysteroid dehydrogenase, along with a reduction in testosterone synthesis, suggests a potential disruption of male gonadal function [98]. In contrast, histological analysis of testes from rats exposed to treatments for 28 days showed normal Sertoli and Leydig cells along with regular arrangement of spermatogenic cells [99], indicating a neutral impact of HTP exposure on the male reproductive system. However, it should be pointed out that the HTPs used in the study were test samples from the New Tobacco Products Engineering and Technology Research Center of Sichuan Province, Chengdu, China, and not devices commercially available.

Further insights on oxidative stress-mediated testis impairment come from OECD 90-day sub-chronic exposure, which showed how both HTP aerosol and CS increased oxidative stress in the testes, as evidenced by an increase in ROS generation and MDA levels along with a reduction in antioxidant capability. Pro-inflammatory factors such as IL-1, IL-6, and TNF were found to be increased in both HTP and CS exposed animals. TNF and interleukins play a key role in coordinating immune cell function and supporting spermatogenesis. However, under certain pathological conditions, elevated levels of pro-inflammatory cytokines can shift the homeostatic balance toward enhanced immune and inflammatory responses. This may lead to the recruitment of immune cells to the testes or their activation within germ cells, potentially resulting in germ cell apoptosis and impaired spermatogenesis [94]. Moreover, the authors reported an increased activation of NLRP3 inflammasome through the ROS/NLRP3/Caspase-1 pathway, leading to pyroptosis, a programmed lytic cell death characterized by rapid plasma membrane rupture [100].

### 5.1. Clinical Data on HTP Impact on the Male Reproductive System

Given that atherosclerotic lesions are often irreversible, the observed improvement in erectile function following smoking cessation is likely attributable primarily to the reduction in oxidative stress. The current literature lacks comparative data on the prevalence of erectile dysfunction among users of e-cigarettes or HTPs versus conventional cigarette smokers or non-smokers, thereby precluding definitive conclusions regarding the impact of these alternative nicotine delivery systems on erectile function. However, a recent systematic review and other original investigations indicate that both e-cigarette and HTP consumption may impair erectile function [101]. This speculation is supported by the fact that nicotine itself is a known vasoconstrictor reported to cause endothelial impairment through oxidative stress and impair sexual and erectile function in animal models and humans [102]. However, to date, there are no relevant clinical data available on the effects of HTP use on erectile dysfunction nor on the potential benefits of switching from conventional cigarettes to HTPs. Similarly, clinical studies investigating the potential effects of HTP use on semen quality are currently lacking.

### 5.2. Clinical Data on HTP Impact on the Female Reproductive System

A recent clinical trial reports that the use of HTPs negatively affected fertility, leading to reduced ovarian quality and ovarian reserve. It had a detrimental impact on both the quality and quantity of oocytes retrieved during intracytoplasmic sperm injection cycles (ICSI). Additionally, users of HTPs exhibited decreased levels of anti-Müllerian hormone (AMH) [103].

In line, pregnant mothers who smoked HTPs were more at risk of preterm birth, similar to cigarette smokers (CS) [104], and those who smoked HTPs exclusively during pregnancy were at higher risk of small for gestational age (SGA) than those who had never smoked [105].

Overall, studies tackling the effect of HTP consumption on reproductive health are not yet sufficient, and no clear conclusion can be drawn on the potential detrimental effects of HTP use on reproduction and offspring health.

## 6. Conclusions and Future Outlook

Given the growing public emphasis on detrimental health effects associated with CS, the shift from conventional cigarette use is rapidly turning into an expanding phenomenon, particularly in industrialized countries, and is especially appealing to younger individuals. While the perception of a less harmful product may play a significant role, the adoption of these devices may also be driven by social imitation and peer influence.

Based on the relevant literature, reviewed here, it is clear that HTPs allow for nicotine inhalation at concentrations very similar to those observed with conventional cigarettes, while exposing users to significantly lower levels of HPHC. On this point, there is substantial agreement in the literature. However, it is equally clear that, based on the reduced HPHC content, it was initially assumed—at least in the early phases—that these devices would be inherently safer. Evidence from basic research studies and clinical trials strongly suggests that exposure to HTPs may contribute to the onset or exacerbation of certain diseases typically associated with cigarette smoking. Notably, the cellular and molecular mechanisms underlying the effects of HTPs often mirror those observed with conventional cigarette smoke exposure, as summarized in Table 4. Current evidence indicates that exposure to HTPs is associated with an increase in oxidative stress, and it represents a key mechanism through which tobacco products impair organ function. In addition, a further common feature of HTP exposure is its pro-inflammatory effect. These two non-negligible aspects are central to the etiology of various smoking-related diseases and indeed form the basis of many of the studies discussed above.

It is important to clearly reiterate that, with few exceptions, comparative studies—including clinical trials—consistently report that the use of HTPs has a lower impact compared to conventional cigarette smoking. This inevitably leads to a discussion on the potential benefit that a smoking patient might derive from switching to HTP use. However, long-term data to support this hypothesis are currently lacking. The mere observation of less pronounced pathological effects in HTP users over the short term does not provide definitive predictive value. Furthermore, HTP users are often considered former smokers, overlooking the possibility that these devices may also serve as a gateway to tobacco use for new users. In this regard, the role of HTPs in smoking cessation is under debate, and recent clinical studies indicate that HTPs fail to help smokers quit or prevent former smokers from relapsing [106,107,108]. This should not come as a surprise, since HTPs mimic traditional cigarettes in terms of nicotine and gestural behavior. Furthermore, the dual-use pattern is often overlooked. While extrapolating current data on HTP-only users remains uncertain, the situation becomes even more complex when considering dual-users.

As a whole, to date, the risk and safety evaluation of HTPs needs further research, in particular:

- While there is a rich and detailed body of literature on the reproductive effects of smoking and fetal development, data regarding the use of HTPs remain very limited.
- Many studies compare the effects of HTPs with conventional smoking (CS) and focus their conclusions on this comparison, often neglecting the investigation of the intrinsic toxicity of the devices themselves.
- An increase in cohort studies, exploring in greater depth, the early warning toxicity markers of exposure as well as epigenetic changes in cancer-regulating genes.

Since we are far from definitively understanding the health impact of HTPs, even compared with cigarettes, there is a need for longer-term, unbiased clinical trials and epidemiological studies to assess: the long-term effects of HTP use under real-world conditions and the health consequences in the increasing population of non-smokers who are adopting these products.

**Table 4:** Key effects associated with the use of heated tobacco products (HTPs) and their corresponding biomarkers.

Health Concern	Molecular Pathways/Biomarkers Involved	Studies
↑ Inflammation, cellular damage, and oxidative stress	↑ Cytokine IL-1β, IL-6 expression ↑ activation of DAMPs ↑ Nrf2 antioxidant response ↑ DNA oxidative damage	In vitro [29]
↑ Lung and tracheal remodeling, inflammation, and oxidative response	↑ NF-κB pathways ↑ Granulocytes pulmonary infiltration ↑ Circulating blood neutrophils ↑ CCL expression ↑ GM-CSF	In vivo [30,31,32]
↑ Lung parenchyma alteration	↑ Lipid accumulation in alveolar macrophages	In vivo [33]
↑ Lung infiltrating leukocytes	↑ CD4^+^ ↑ RORγt^+^ receptor ↑ CXCL8	In vivo [33]
↑ Increased risk of atherosclerosis, oxidative stress, and endothelial dysfunction	↓ Glutathione ↑ Inflammatory markers ↑ Monocyte adhesion ↑ TNF-α ↑ IL-1β ↑ Monocyte adhesion	In vitro [40,41]
↑ Endothelial dysfunction	↓ Phosphorylation of eNOS at Ser1177 ↓ eNOS ↓ NO generation	In vitro [42]
↓ COPD flare-up	↓ Exacerbation rates ↓ Respiratory symptoms	Clinical data [44]
↑ Risk of acute eosinophilic pneumonia (AEP)	↑ Eosinophils infiltration ↑ Allergic reaction	Clinical data [74,75]
↑ Risk of asthma ↑ Allergic rhinitis ↑ Atopic dermatitis		Clinical data [76]
↓ Cardiovascular risk	↑ HDL-C ↓ White blood cell (WBC) ↑ Forced expiratory volume ↓in one second (FEV1) ↓ Carboxyhemoglobin (COHb) ↓ Total 4-(methylnitrosamino)-1-(3-pyridyl)-1-butanol (Total NNAL)	Clinical data [58]
↓ COPD-associated symptomatology	↓ COPD exacerbation rate	Clinical data [73]
↓ Oxidative stress	↓ Carboxyhemoglobin (COHb)	Clinical data [77]
↓ Endothelial function ↑ Elevated oxidative stress ↑ Enhanced platelet activation	↑ Nox2-derived peptide (sNox2-dp) ↑ Serum hydrogen peroxide (H_2_O_2_) levels ↓ NO bioavailability ↓ Flow-mediated dilatation (FMD)	Clinical data [60]
↑ Cardiovascular risk	↓ Systolic and diastolic myocardial function	Clinical data [78]
↑ Cardiovascular risk	↑ Plasma biomarkers of glutamate metabolism (i.e., glutamate, arginine, ornithine, and citrulline).	Clinical data [68]
↑ Cardiovascular risk and endothelial dysfunction	↑ Arterial vascular stiffness ↑ Platelet thrombus formation	Clinical data [79,80]
↑ Cortical vessel dilatation	↑ Nicotine-induced α4β2-nAChR activation	In vivo [82]
↑ Oxidative stress ↑ Neuroinflammation	↑ Nrf2 antioxidant response ↑ Microglia activation	In vitro [84,109]
↑ Oxidative stress ↑ Neuroinflammation	↑ ROS production ↑ PPARα and PPARγ nuclear receptors ↑ 8-hydroxyguanosine ↑ Xeroderma pigmentosum group C protein complex (XPC) ↑ 8-oxoguanine DNA glycosylase-1 (OGG-1)	In vivo [85]
↑ Alzheimer’s disease risk	↑ Mesolimbic dopaminergic neurotransmission	Clinical data [78]
↑ Neuroinflammation	↑ KDM6B ↓ PPARα and PPARγ PTEN ↑ AKT	In vivo [89]
↑ Psychiatric disorders risk	↑ Anhedonia ↑ Depressed mood	Clinical data [92]
↓ Male gonadal function	↓ Spermatogenesis ↑ Sexual maturation time	In vivo [96]
↓ Male gonadal function ↓ Testosterone levels ↑ Oxidative stress	↑ NF-κB ↑ TNF-α ↑ IL-1β ↑ IL-6, ↑ COX-2 ↓ 3β-hydroxysteroid dehydrogenase ↓ 17β-hydroxysteroid dehydrogenase	In vivo [98]
↓ Male gonadal function ↑ Inflammation ↑ Oxidative stress	↑ ROS ↑ MDA ↑ IL-1 ↑ IL-6 ↑ TNF	In vivo [94]
↓ Female fertility	↓ Anti-Müllerian hormone (AMH) ↓ Quality and quantity of oocytes retrieved during Intracytoplasmic sperm injection cycles (ICSI)	Clinical data [103]
↓ Female fertility	↑ Preterm birth ↑ Risk of small for gestational age (SGA)	Clinical data [104,105]

## Figures and Tables

**Table 1:** Comparative review of the main HPHCs reported in the literature for HTP mainstream and traditional cigarette (TC) smoke.

HPHC	Mainstream HTP	TC Smoke
Nicotine (mg)	0.3–1.5	0.7–2.1
Formaldehyde (µg)	0.9–22.6	3.2–74.4
Acetaldehyde (µg)	128.5–301. 5	567.0–1534
Acetone (µg)	18.8–48.37	95.5–775.6
Acrolein (µg)	0.9–13.1	1.1–160.9
Propionaldehyde (µg)	7.8–22.3	29.6–124.0
Crotonaldehyde (µg)	0.7–6.4	10.10–65.7
Methacrolein (µg)	6.5	85.5
Butyraldehyde (µg)	14.9–30.7	22.2–65.0
Valeraldehyde (µg)	20.1	-
Glyoxal (µg)	3.1	-
Methyl glyoxal (µg)	33.5	-
2-Butanone (µg)	4.2–6.5	11.0–220.5
Polycyclic aromatic hydrocarbon (PAH)		
Benzo(a)pyrene (ng)	−0.8	6.7–20
Naphthalene	1.6	1105
Acenaphthylene (ng)	1.9	235
Acenaphthene (ng)	145	49
Fluorene (ng)	1.5	371
Anthracene (ng)	0.3	130
Phenanthrene (ng)	2.0	292
Fluoranthene (ng)	7.3	123
Pyrene (ng)	6.4	89
Benz[a]anthracene (ng)	1.8	33
Chrysene (ng)	1.5	48
Benzo[b]fluoranthene(ng)	0.5	24
Benzo[k]fluoranthene(ng)	0.4	4.3
Total particulate matter	12.9–55.8	9.8–37.7
Tar	7.5–16.6	8.0–25.50
Carbon monoxide	0.3–0.5	2–33.0

Range of harmful and potentially harmful constituents (HPHC) per stick or tobacco cigarette are reported for heated tobacco product mainstream (HTP) and traditional tobacco cigarette (TC smoke). The value ranges derived from the following studies: [16,18,19,20,21,22,23,24,25]. The puffing regimen used in the different studies is summarized below: International Organization for Standardization—ISO 3308:2000 (ISO): puff volume: 35 mL; duration: 2 s; interval: 60 s; six puffs per HTP stick; six puffs per TC. Health Canada Intense puffing regimens—T-115 (HCI): puff volume: 55 mL; duration: 2 s; interval: 30 s; 12 puffs per HTP stick; 8–12 puffs per TC. Cooperation Center for Scientific Research Relative to Tobacco—N°64 (CORESTA): puff volume: 75 mL; duration: 2.5 s; interval: 30 s; 7–12 puffs per HTP stick; 11 puffs per TC. The study by Auer et al. followed International Organization for Standardization standards for puff volume (35 mL) at 2 puffs per minute, based on observation of HTP users who took a mean of 14 puffs during 5 to 6 min. (-). The symbol indicates that the marker was not reported or below the detection level. TC smoke data were obtained from reference-grade (3R4F, 1R6F) cigarettes or Marlboro Red cigarettes.

**Table 2:** Approximate fold increase in HPHCs in HTP compared to 3R4F reference cigarette smoke (CS).

HPHC	Fold Increment Compared to 3R4F Smoke
1,4-Dioxane, 2-ethyl-5-methyl-	137
Hexadecanoic acid, ethyl ester	60
Trans-4-hydroxymethyl-2-methyl-1,3- dioxolane	47
Stearate, ethyl-	24
12,14-Labdadiene-7,8-diol, (8a,12E)	21
Butylated hydroxytoluene	18
Ethyl linoleate	16
Labdane-8,15-diol, (13S)	9
Propylene glycol	6
2-Furanmethanol	4
Butyrolactone	5
Methyl furoate	4
2-Cyclopentene-1,4-dione	4
2-Furanmethanol, 5-methyl-	3
2-Cyclopentene-1,4-dione	3
2-Methylcyclobutane-1,3-dione	3
Lanost-8-en-3-ol, 24-methylene-, (3beta)	3
2-Furancarboxaldehyde, 5-methyl-	3
Eicosane, 2-methyl-	3
1,2,3-Propanetriol, diacetate (diacetin)	2
Glycidol	2
Heneicosane, 2-methyl-	2

Data adapted from St Helen et al., 2018, and Upadhyay et al., 2023 [25,28]. The present list represents an extract of HPHCs recording to be more than 2-fold higher in HTP streaming compared to TC smoke.

**Table 3:** Key evidence from clinical studies on HTP use.

Authors	Nicotine Delivery System	Study Design	Intervention Period	Key Findings	Main Redox Biomarkers
Polosa et al., 2021 [44]	Switch from CS to HTPs	COS	3 years	↓ COPD exacerbations ↑ CAT score ↑ 6 MWD	carbon monoxide carboxyhemoglobin
Roethig et al., 2005 [45]	Switch from CS to HTPs	RCT	8 days	↓ Urine mutagenicity	↓ carbon monoxide ↓ carboxyhemoglobin
Roethig et al., 2007 [46]	Switch from CS to HTPs	RCT	8 days	↓ Urine mutagenicity	↓ carbon monoxide ↓ carboxyhemoglobin
Tricker et al., 2012 [47]	Switch from CS to HTPs vs. CS	RCT	6 days	↓ Excretion of mutagenic material in urine	↓ carbon monoxide ↓ carboxyhemoglobin
Tricker et al., 2012 [48]	Switch from CS to HTPs vs. CS	RCT	8 days		
Martin Leroy et al., 2012 [49]	Switch from CS to HTPs vs. CS	RCT	35 days	↓ WBC count ↓ 11-DTXB_2_	↓ carboxyhemoglobin ↓ *hs*-CRP
Sakaguchi et al., 2014 [50]	Switch from CS to HTPs	RCT	28 days	↓ Urine mutagenicity	- carboxyhemoglobin
Shepperd et al., 2015 [51]	Switch from CS to reduced-toxicant-prototype cigarette (RTP)	RCT	6 months	↓ Urine mutagenicity ↓ 2-cyanoethylvaline hemoglobin adducts - 4-ABP hemoglobin adducts ↓ 11-DTXB_2_ ↑ s-ICAM-1	- 8-iso-PGF2α type III
Ogden et al., 2015 [52]	Switch from CS to HTPs	RCT	24 weeks	↓ 4-ABP hemoglobin adducts	- carboxyhemoglobin ↑ 3-HPMA
Haziza et al., 2016 [53]	Switch from CS to HTPs vs. CS	RCT	5 days	↓ S-PMA ↓ MHBMA ↓ 3-OH-B[a]P ↓ CYP1A2 activity	↓ carboxyhemoglobin ↓ 3-HPMA
Haziza et al., 2017 [54]	Switch from CS to HTPs vs. CS	RCT	5 days	↓ S-PMA ↓ MHBMA ↓ 3-OH-B[a]P ↓ 4-ABP hemoglobin adducts	↓ carboxyhemoglobin ↓ 3-HPMA
Ludicke et al., 2017 [55]	Switch from CS to HTPs vs. CS	RCT	5 days	↓ S-PMA ↓ MHBMA ↓ 3-OH-B[a]P ↓ 4-ABP hemoglobin adducts	↓ carboxyhemoglobin ↓ 3-HPMA
Ludicke et al., 2018 [56]	Switch from CS to HTPs vs. CS	RCT	85 days	↓ sICAM-1 ↓ 11-DTX-B2 ↑ FEV_1_ ↓ hs-CRP ↓ WBC count	↓ 8-epi-PGF2α
Gale et al., 2019 [57]	Switch from CS to HTPs vs. CS	RCT	6–7 days	↓ S-PMA ↓ MHBMA ↓ 4-ABP hemoglobin adducts	↓ 3-HPMA
Ludicke et al., 2019 [58]	Switch from CS to HTPs vs. CS	RCT	6 months	↓ WBC count ↑ FEV_1_ ↑ HDL-c	↓ carboxyhemoglobin -8-epi-PGF2α
Haziza et al., 2020 [59]	Switch from CS to HTPs vs. CS	RCT	90 days	-Mutagenicity (Ames test) ↓ CYP1A2 activity ↓ S-PMA ↓ MHBMA ↓ 4-ABP hemoglobin adducts	↓ carboxyhemoglobin ↓ 3-HPMA
Loffredo et al., 2021 [60]	Switch from CS to HTPs vs. CS	RCT	3 years	-PA -sCD40L -sP-selectin -FMD -NO	↑ -hydrogen peroxide ↓ sNox2-dp
Sakaguci et al., 2021 [61]	Switch from CS to HTPs vs. CS	COS	28 days	↑ HDL-c ↓ sICAM-1 ↓ WBC count ↓ 11-DTX-B2 - FEV_1_	↓ 8-epi-PGF2α
Ikonomidis et al., 2021 [62]	Switch from CS to HTPs vs. CS	COS	1 month	↓ PWV ↓ brachial systolic ↓ blood pressure ↓ heart rate ↑ FMD ↑ CFR ↑ GWI ↑ GWW ↓ GWE	↓ MDA ↓ TxB2 ↓ PC ↓ exhaled carbon monoxide
Gale et al., 2021 [63]	Switch from CS to HTPs vs. CS	RCT	6 months	↓ WBC count ↑ FeNO ↑ HDL ↓ 11-DTX-B2 ↓ sICAM-1 ↑ FEV_1_	↓ 8-epi-PGF2α
Ohmomo et al., 2021 [64]	Switch from CS to HTPs vs. CS	COS	<2 years	↓ hypomethyleation of *AHRR*, *F2RL3*, and *RARA* genes *↓ AHRR* gene expression	
Gale et al., 2022 [65]	Switch from CS to HTPs vs. CS	RCT	1 year	↓ MHBMA ↓ S-PMA ↓ 4-ABP hemoglobin adducts ↑ FeNO ↓ WBC count ↓ 11-DTX-B2 ↑ HDL-c	↓ 3-HPMA -8-epi-PGF2α ↓ exhaled carbon monoxide
Nishihara et al., 2023 [66]	Switch from CS to HTPs vs. CS	RCT	5 days	↓ C_max_ ↓ AUC_0–tlast_ ↓ mCEQ subscale of “Smoking Satisfaction”	
Li et al., 2023 [67]	Switch from CS to HTPs vs. CS	RCT	28 days	↓ VOCs urine concentration	↓ 3-HPMA
Harada et al., 2024 [68]	Switch from CS to HTPs vs. CS	COS	3 years	-glutamate metabolism	
Spicuzza et al., 2024 [69]	Switch from CS to HTPs vs. CS	RCT	12 weeks	↑ V̇O2max maximal oxygen consumption	↓ carboxyhemoglobin ↓ exhaled carbon monoxide
Ansari et al., 2024 [70]	Switch from CS to HTPs vs. CS	RCT	12 months	↑ HDL-c ↓ 11-DTX-B2 ↓ sICAM-1 ↑ FEV_1_ ↓ WBC count	↓ 8-epi-PGF2α ↓ myeloperoxidase ↓ *hs*-CRP
Lyytinen et al., 2024 [71]	HTP users vs. non-exposed subjects	COS	1 day	↑ PWV ↑ AIx75 ↑ platelet-dependent thrombus formation	
Chu et al., 2025 [72]	Switch from CS to HTPs vs. CS	RCT	21 days	↓ MHBMA ↓ CEMA	↓ 3-HPMA ↓ exhaled carbon monoxide

The studies summarized in the table represent the principal clinical trials retrieved using the following search string within the PubMed database: [“heated tobacco” OR “heat-not-burn” OR “tobacco heating” OR “unburned cigarette” OR “IQOS” OR “Ploom” OR “glo”) AND “biomarker” OR “trial”]. For the purposes of the present review, we included studies that investigated biomarkers of exposure and biological effects associated with the use of heated tobacco products (HTPs). Studies specifically addressing smoking cessation were not considered in this discovery. Upward and downward arrows indicate an increase or a decrease in the biomarker, respectively, whereas a dash indicates that no significant change was observed. The following acronyms are used in order of appearance COS: Cohort study; RCS: Randomized controlled study; CAT score: changes in daily cigarette smoking, annualized disease exacerbations, lung function indices, patient-reported outcomes; 6 MWD: 6 min walk distance; hs-CRP: high sensitivity C-reactive protein; 11-DTXB_2_: 11-dehydro-thromboxane B2; 8-iso-PGF2α: 8-iso Prostaglandin F2α; s-ICAM-1: soluble intercellular adhesion molecule 1; N-acetyl-S-3-hydroxypropylcysteine; S-PMA: S-phenylmercapturic acid; MHBMA: monohydroxybutyl mercapturic acid; FEV_1_: forced expiratory volume in 1 s; sNox2-dp:soluble Nox2-derived peptide; PA: plaquet-activated; sCD40L:soluble CD40 ligand; FMD: flow-mediated dilation; VOCs: volatile organic compounds; PWV: Carotid-femoral pulse wave velocity; CFR: coronary flow reserve; PC: protein carbonyls; GWI: myocardial work index; GWW: global wasted myocardial work; GWE: global myocardial work efficiency; FeNO: fractional exhaled nitric oxide; VO2max: maximum oxygen level consumed; AIx75: augmentation index corrected for heart rate; CEMA: 2-cyanoethylmercapturic acid.

## Data Availability

Data supporting the findings of this study are available upon reasonable request.
